# The influence of physical activity during pregnancy on maternal, fetal or infant heart rate variability: a systematic review

**DOI:** 10.1186/s12884-016-1121-7

**Published:** 2016-10-26

**Authors:** Pavel Dietz, Estelle D. Watson, Matteo C. Sattler, Wolfgang Ruf, Sylvia Titze, Mireille van Poppel

**Affiliations:** 1 Department of Physical Activity and Public Health, Institute of Sports Science, University of Graz, Mozartgasse 14, Graz, 8010 Austria; 2 Centre for Exercise Science and Sports Medicine, School of Therapeutic Sciences, Faculty of Health Sciences, University of Witwatersrand, Private Bag 3, Johannesburg, 2050 South Africa; 3 Department of Public and Occupational Health, EMGO Institute for Health and Care Research, VU University Medical Centre, Van der Boechorststraat 7, Amsterdam, 1081 BT The Netherlands

**Keywords:** Pregnancy, Public health, Heart rate variability, Childbirth, Stress, Offspring

## Abstract

**Background:**

Physical activity (PA) during pregnancy has been shown to be associated with several positive effects for mother, fetus, and offspring. Heart rate variability (HRV) is a noninvasive and surrogate marker to determine fetal overall health and the development of fetal autonomic nervous system. In addition, it has been shown to be significantly influenced by maternal behavior. However, the influence of maternal PA on HRV has not yet been systematically reviewed. Therefore, the aim of this systematic review was to assess the influence of regular maternal PA on maternal, fetal or infant HRV.

**Methods:**

A systematic literature search following a priori formulated criteria of studies that examined the influence of regular maternal PA (assessed for a minimum period of 6 weeks) on maternal, fetal or infant HRV was performed in the databases Pubmed and SPORTDiscus. Quality of each study was assessed using the standardized Quality Assessment Tool for Quantitative Studies (QATQS).

**Results:**

Nine articles were included into the present systematic review: two intervention studies, one prospective longitudinal study, and six post-hoc analysis of subsets of the longitudinal study. Of these articles four referred to maternal HRV, five to fetal HRV, and one to infant HRV. The overall global rating for the standardized quality assessment of the articles was moderate to weak. The articles regarding the influence of maternal PA on maternal HRV indicated contrary results. Five of five articles regarding the influence of maternal PA on fetal HRV showed increases of fetal HRV on most parameters depending on maternal PA. The article referring to infant HRV (measured one month postnatal) showed an increased HRV.

**Conclusions:**

Based on the current evidence available, our overall conclusion is that the hypothesis that maternal PA influences maternal HRV cannot be supported, but there is a trend that maternal PA might increase fetal and infant HRV (clinical conclusion). Therefore, we recommend that further, high quality studies addressing the influence of maternal PA on HRV should be performed (methodological conclusion).

## Background

During pregnancy, maternal physiological systems progressively adapt to accommodate the increasing demands of fetal growth and development [1]. This includes an increase in cardiovascular and respiratory output, an increase in renal function as well as endocrine and metabolic changes [2–4]. Due to these changes, pregnancy is a ‘stress test’ for the metabolic and physiologic system of women, and can lead to pregnancy-induced complications and adverse health effects. Pregnancy associated conditions such as gestational diabetes, hypertension, mental disorders and maternal obesity have been shown to alter fetal development and increase the risk of diseases in the offspring and the infant’s later life [5–8]. Furthermore, the risk for some of these maternal adverse health effects has been shown to be associated with increased sympathetic activity during gestation [9].

One method to monitor sympathetic and parasympathetic control of the autonomic nervous system (ANS) is heart rate variability (HRV). It describes beat-to-beat variation in cardiac R-R interval. Analyzing these R-R intervals in different frequency and time domains provides information on whether changes in heart rate (HR) are mediated primarily by the sympathetic or parasympathetic arm of the ANS [10, 11]. In adults, increased HRV has been associated with higher aerobic fitness and may therefore lead to possible maternal health benefits [12]. Furthermore, several studies indicated that exercise has positive effects on HRV in both healthy adults and children [13, 14]. With regard to the influence of overweight and obesity (OaO) on HRV, increased BMI has been associated with reduced HRV and increased sympathetic and decreased parasympathetic activity [15–18]. Part of this might be due to reduced physical activity (PA) levels in people with higher BMI, since in OaO adults who were regularly physically active HRV was higher compared to sedentary peers [19].

In healthy pregnant women, sympathetic control also increases with progressing gestation as consequence of physiological adaptations of the maternal body, resulting in an altered HRV compared to healthy non-pregnant women [20]. In the growing fetus, HRV is used as a surrogate marker to determine the development of fetal ANS and overall fetal health [11, 21, 22]. However, fetal HRV is strongly influenced by maternal behavior (e.g. cigarette smoking and alcohol consumption) [23, 24].

Throughout pregnancy, regular PA, which is defined as ‘any bodily movement produced by skeletal muscles that results in energy expenditure’ [25], has been shown to improve maternal cardiovascular and respiratory output, improve insulin resistance and metabolic control, reduce the risk of gestational diabetes mellitus as well as depressive symptoms [26–31]. In non-pregnant women, adult men as well as in children, PA has further been shown to affect cardiovascular autonomic control and HRV [32–35].

Although HRV is a noninvasive method to monitor control of ANS of the mother as well as overall fetal development, the influence of maternal PA on HRV has not yet been systematically reviewed. Therefore, within the present article we systematically reviewed studies that assessed the influence of PA during pregnancy on maternal, fetal or infant HRV. Based on the review, a more definitive conclusion on the influence of maternal PA on maternal, fetal and infant HRV can be obtained. In addition, recommendations for future studies in this field will be formulated.

## Methods

### Literature search

A systematic literature search of studies that examined the influence of regular maternal PA on maternal, fetal or infant HRV was performed in the databases Pubmed and SPORTDiscus. Therefore, a search term was created that combines ‘physical activity’, ‘pregnancy’, and ‘cardiovascular outcomes’. The final search term for Pubmed can be found in Appendix 1. For the search on SPORTDiscus, this search term was adapted following the SPORTDiscus search guidelines. Since the search term was very specific, no further limits were used to specify search. The search was completed on February 2^nd^ 2016.

### Inclusion criteria and selection process

In this systematic review, all studies had to fulfil the following inclusion criteria: (1) assessing the influence of PA during pregnancy on HRV in (a) mother, (b) fetus or (c) infant; (2) dealing with human subjects; (3) dealing with any kind of PA (e.g. aerobic exercise, resistance exercise, yoga, gymnastics, etc.); from a public health point of view, the effects of regular PA and not the effects of acute PA are of interest, consequently (4) PA levels should be assessed for a minimum period of 6 weeks; (5) abstracts had to be available in English, German or Dutch language; (6) only full papers published in peer-reviewed journals were considered. Since the aim of this systematic review is providing an overview of all studies assessing HRV dependent on PA in pregnancy, (7) all study designs were included (e.g. prospective studies, retrospective studies, intervention studies, observational studies etc.) in order to draw an overall conclusion.

**Fig. 1:**
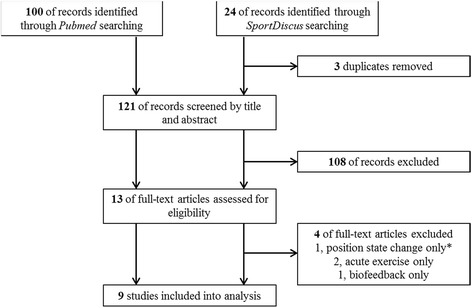
Flow chart of the literature search. *‘Position state change only’ means that the only measured activity was the change from sitting to standing position

Altogether, 124 articles were identified (Fig. 1). After removing three duplicates, 121 articles were screened by abstract and title for possible inclusion. Of these, 108 articles were excluded due to not passing all of the a priori formulated criteria. Finally, full texts of 13 articles were screened [34, 36–47]. Of these, 4 were excluded for specific reasons (Fig. 1) so that nine articles were included into the final systematic review.

### Quality assessment and strength of evidence

For assessing the quality of the included articles, the standardized Quality Assessment Tool for Quantitative Studies (QATQS) [48], a well-established and validated tool for assessing the quality of studies in the field of public health research [49, 50], was used. It consists of six components: (1) the extent to which the participants are representative of the target population (selection bias), (2) study design, (3) control of confounding factors, (4) blinding of outcome assessors and participants, (5) reliability and validity of the data-collection tools, and (6) the number of and reasons for withdrawals and drop-outs. The component ‘blinding of outcome assessors and participants’ was considered not applicable for observational and intervention studies in this review. The reason for considering ‘blinding’ not applicable for intervention studies in this case is that in studies with PA intervention the assessors (i.e. researchers) and the participants are very likely to know the outcome of the randomization [51]. According to standardized criteria, each component (1-6) of each article was rated as ‘strong’, ‘moderate’ or ‘weak’. Finally, an overall rating was calculated on the basis of the individual ratings also following standardized criteria: the overall rating was ‘strong’, when there was no ‘weak’ component rating, ‘moderate’ when there was only one ‘weak component rating, and ‘weak’ when there were two or more ‘weak’ component ratings. A detailed description of, as well as the criteria for, the six component ratings can be found online [48].

### Data extraction

The systematic literature search using the above described search terms as well as the a priori formulated inclusion criteria has been performed by PD and was checked by a second researcher (ST). Of the nine included articles, all relevant data were extracted by PD following a commonly used table format including study design, sample and subjects, methods, outcomes, statistics. Extracted data were checked by two researchers (EW, MvP). Quality assessment for each study using the QATQS has been performed by two researchers independently (PD, MS). When researches came to different results, these points were discussed within the research team to come to a consensus.

## Results

### Study descriptions

Nine articles were included into the present systematic review of which three refer to maternal HRV, four to fetal HRV, one to both maternal and fetal HRV, and one to infant HRV. In summary, four articles provide results on maternal HRV, five on fetal HRV, and one on infant HRV. Of these, two articles were intervention studies [36, 43]. The intervention study performed by Stutzman et al. [43] assessed the effects of low intensity exercise in normal weight (NW) and overweight and obese (OaO) pregnant women on maternal HRV. The intervention study performed by Satyapriya et al. [36] assessed the effect of yoga specifically designed for the second and third trimester of pregnancy, compared to standard prenatal exercise (intensity not specified) on maternal HRV. One article was a prospective longitudinal study which was designed to determine the influence of self-reported maternal exercise on fetal cardiac ANS development [40]. To assess self-reported maternal exercise, the authors used the ‘Modifiable Physical Activity Questionnaire’ (MPAQ). Finally, the remaining six articles [37–39, 41, 42, 47] were post-hoc analyses of subsets of the before mentioned longitudinal study of May et al. [40]. Four of these post-hoc analyses referred to maternal or fetal HRV measurements performed in gestational week 36, one to maternal HRV measurements performed at 28, 32, and 36 weeks of gestation, and one to infant HRV measurements at one month of age. The different HRV parameters assessed in the nine studies as well as their physiological association are summarized and described in Table 1. The cohort and study characteristics are summarized in Table 2.

**Table 1:** HRV parameters and their physiological association

Parameter	Abbreviation	Physiological association
Time domain		
Standard deviation of NN intervals	SDNN	Overall HRV, sympathetic and parasympathetic innervation
Root mean square of successive difference between NN intervals	RMSSD	Short-term HRV, primarily parasympathetic innervation
Frequency domain		
Total power	TP	Total HRV (as the band encompasses all frequencies)
High frequency	HF	Parasympathetic control
Ratio high frequency and total power	HF/TP	Parasympathetic control
Intermediate frequency	IntF	Sympathetic and parasympathetic arm of the ANS
Low frequency	LF	Sympathetic and parasympathetic arm of the ANS
Very low frequency	VLF	Sympathetic control
Ratio very low frequency and high frequency	VLF/HF	Sympatho-vagal balance
Ratio very low frequency and low frequency	VLF/LF	Sympatho-vagal balance
Ratio low frequency and high frequency	LF/HF	Sympatho-vagal balance

(*ANS* autonomic nervous system, *NN* normal-to-normal)

**Table 2:** Cohort and study characteristics

First author, year	Study design	Sample (pregnant women)	Intervention, exercise mode, PA determination	HRV outcomes (maternal/fetal/infant)	HRV measure technique	Time(s) of measurement	Data analysis
May, [47]	Post-hoc analysis of maternal magnetocardiograms (May et al. [40]) to assess maternal heart measures depending on maternal exercise behavior (exercise vs. control group) at different weeks of gestation	*N* = 56 (gw36 *N* = 51); 20–39 years singleton, low-risk pregnancies	MPAQ to retrospectively categorize women into exercise (≥30 min. aerobic exercise/ 3 times a week) and control group^a^	maternal M: SDNN, RMSSD, VLF, LF, HF, LF/HF	3 continuous, 18 min simultaneous fetal-maternal MCG were recorded for each subject using an investigational 83-channel dedicated fetal biomagnetometer	Gw28, gw32, and gw36	Students *t*-test and Wilcoxon rank sum test to contrast variables between exercise and control groups
Van Leeuwen, [41]	Post-hoc analysis of fetal-maternal magnetocardiograms (May et al. [40]) to assess maternal and fetal heart measures depending on maternal exercise behavior (exercise vs. control group)	*N* = 40; 20–35 years singleton, low-risk pregnancies	MPAQ to retrospectively categorize women into exercise (≥ 30 min. of moderate to vigorous aerobic exercise/ 3 times a week; *n* = 21) and control group (*n* = 19)^a^	maternal, fetal M: SDNN, RMSSD F: SDNN, RMSSD	A continuous, 18 min simultaneous fetal-maternal MCG was recorded for each subject using an investigational 83-channel dedicated fetal biomagnetometer	Gw36	Comparison between control and exercise groups using Mann-Whitney-U test
May, Suminski, [37]	Post-hoc analysis of fetal magnetocardiograms (May et al. [40]) to assess fetal heart measures depending on the duration of maternal continuous and non-continuous leisure-time physical activity	*N* = 40; 23–39 years singleton, low-risk pregnancies	MPAQ to retrospectively classify whether the women perform continuous (e.g., walking, jogging) or non-continuous (e.g. weight lifting, yoga)^a^ leisure-time physical activity	fetal F: SDNN, RMSSD, VLF, LF, HF	A continuous, 18 min simultaneous fetal-maternal MCG was recorded for each subject using an investigational 83-channel dedicated fetal biomagnetometer	Gw36	Pearson Product Monument correlation to assess relationships between duration (min) of maternal continuous and non-continuous LTPA and fetal heart measures; Multiple regression analyses to predict fetal heart measures
May, Scholtz, [39]	Post-hoc analysis of a subset of infants from a prospective longitudinal pregnancy study (May et al. [40]) to assess infant heart measures depending on maternal exercise behavior (exercise vs. control)	*N* = 43; 20–35 years, singleton, low-risk pregnancies	MPAQ to retrospectively categorize infants of women who were in the exercise (≥ 30 min. of moderate to vigorous aerobic exercise/ 3 times a week; *n* = 16) and control group (*n* = 27)^a^	infant I: SDNN, RMSSD, LF, HF	A continuous, 18 min simultaneous fetal-maternal MCG was recorded for each subject using an investigational 83-channel dedicated fetal biomagnetometer	One month of age	Student´s *t*-tests to compare infant HRV measures between exercise and control group
Gustafson, [42]	Post-hoc analysis of fetal magnetocardiograms (May et al. [40]) to assess fetal heart measures depending on maternal exercise (exercise vs. control)	*N* = 30; 20–35 years, singleton, low-risk pregnancies	MPAQ to retrospectively categorize women into exercise (≥ 30 min. of moderate to vigorous aerobic exercise/ 3 times a week; *n* = 15) and control group (*n* = 15)^a^	fetal F: RMSSD, VLF, LF, LF/HF, VLF/LF, VLF/HF, INT	A continuous, 18 min simultaneous fetal-maternal MCG was recorded for each subject using an investigational 83-channel dedicated fetal biomagnetometer	Gw36	Mixed-effects models with post-hoc comparisons. Comparisons for exercise vs. control were adjusted for breathing patterns (breathing vs. apnea)
May, [38]	Post-hoc analysis of fetal magnetocardiograms (May et al. [40]) to assess effects of maternal exercise dose on fetal heart measures	*N* = 50; 23–39 years, singleton, low-risk pregnancies	MPAQ to retrospectively assess maternal physical activity behavior, specifically duration and intensity^a^	fetal F: SDNN, RMSSD, VLF, LF, HF, VLF/LF, VLF/HF, LF/HF	A continuous, 18 min simultaneous fetal-maternal MCG was recorded for each subject using an investigational 83-channel dedicated fetal biomagnetometer	Gw36	Spearman correlations were used to assess relationships between maternal LTPA (intensity, duration) during third trimester and fetal heart measures Multiple regression were additionally performed to assess how well LTPA intensity and duration predict fetal heart measures
Stutzman [43]	Prospective controlled interventional study to assess pre-to-post changes of exercise conditioning on HRV in normal and overweight women. HRV was measured in supine position and during low-intensity exercise	*N* = 22; healthy, singleton pregnancy; *n* = 10 NW (BMI < 25.0 kg/m^2^), *n* = 12 OaO^b^(BMI > 25.0 kg/m^2^)	Measurements: laboratory testing at gw20 ± 2 and gw36 Intervention: E: Instructed on a16 week progressive low-intensity walking exercise^c^, 5 days/week; *n* = 6 OaO, *n* = 5 NW C: Activity log only; *n* = 6 OaO, *n* = 5 NW →4 group design: a) walking/NW, b) non-walking/NW, c) walking/OaO, d) non-walking/OaO	maternal M: LF, HF, TP, HF/TP, LP/HP	Beat-by-beat R-R intervals were obtained and recorded continuously during testing using three latex-free standard surface ECG electrodes and a Spacelab 514 cardiac monitor	Gw20 and gw36	ANOVAs were used to obtain within and between group changes from gw20 to gw36
May, [40]	Prospective, longitudinal, non-blinded study to assess fetal heart measures depending on self-reported maternal exercise and fetal state	*N* = 61; 20–35 years singleton, low-risk pregnancies	MPAQ to retrospectively categorize women into exercise (≥ 30 min. of moderate to vigorous aerobic exercise/ 3 times a week; *n* = 26) and control group (*n* = 35)^a^	fetal F: SDNN, RMSSD, VLF, LF, IntF, HF	3 continuous, 18 min simultaneous fetal-maternal MCG were recorded for each subject using an investigational 83-channel dedicated fetal biomagnetometer	Gw28, gw32, and gw36	Post-hoc comparisons to obtain changes in fetal heart outcomes between exercise and control groups depending on fetal state (active vs. passive) at gw28, gw32, and gw36
Satyapriya [36]	Prospective, RCT to assess the influence of yoga on maternal HRV (two-group, pre-post-design). Measurements were changes in HRV pre and during exercise session as well as pre and post exercise session (acute effects) at gw20 and gw36 (effects over intervention time; not tested for significance)	*N* = 90; 20–35 years; mean BMI: 25.1 (E) and 25.6 (C)	Intervention: E: two modules of integrated yoga, specifically designed for the second and third trimester of pregnancy (*n* = 45) C: standard prenatal exercise (*n* = 45)	Maternal M: LF, HF, LF/HF	Electrocardiogram was recorded continuously for 5 min before, 10 min during and 5 min after intervention	Gw20 and gw36 Measuring pre, during, and post each session	ANOVAs to obtain within and between group differences in maternal HRV between exercise and control group

(*C* control group, *E* exercise group, *ECG* electrocardiograph, *F* fetal, *gw* gestational week, *HF* high frequency, *HRV* heart rate variability, *I* infant, *IntF* intermediate frequency, *LF* low frequency, *M* maternal, *MCG* magnetocardiogram, *MPAQ* Modifiable Physical Activity Questionnaire, *NW* normal weight, *OaO* overweight and obese, *RMSSD* root mean square of successive difference, *SDNN* standard deviation of normal-to-normal intervals, *TP* total power, *VLF* very low frequency)

^a^The MPAQ was used to retrospectively assess all leisure-time physical activities (LTPA) performed during the last 9 months of pregnancy, plus 3 months before pregnancy (self-reported). Note that no exercise intervention was performed within the highlighted studies

^b^pre-pregnancy BMI; pre-pregnancy weights of the participants were obtained by self-report from the women and confirmed from medical records

^c^the steady-state testing protocol involved a 3-min warm-up at 20 W, followed by a ramp increase in work rate with 30 s to a level corresponding to 40 % of the maximal heart rate reserve

The HRV outcomes depending on PA of the two intervention studies from pre to post, the longitudinal study, as well as of the six post-hoc analyses are presented in Tables 3, 4, and 5. It has to be stressed that for the longitudinal study as well as for the six post-hoc analyses the MPAQ had been used to assess leisure-time PA (LTPA) performed during the 9 months of pregnancy plus 3 months before pregnancy. On the basis of these self-reports, the sample(s) of pregnant women were characterized into exercise or control group (more or less maternal PA) or continuous or non-continuous LTPA group. LTPA had not been objectively measured using, for example, accelerometers in any of the included articles.

**Table 3:** Outcome measurements of each study

First author, year	Type of analyses	SDNN (ms)	RMSSD (ms)	TP (ms^2^)
May, [47]	Difference in maternal HRV between exercise and control group at gw28, gw32, and gw36	maternal gw28: exercise: 53.9 ± 27.4 control: 38.7 ± 12.9 (*p* = 0.01)	maternal gw28: exercise: 40.2 ± 34.1 control 25.9 ± 13.5 (*p* = 0.05)^d^	---
		gw32: exercise: 53.0 ± 19.8 control: 38.1 ± 13.7 (*p* = 0.002)	gw32: exercise: 32.7 ± 24.9 control: 22.9 ± 11.7 (n.s.)	
		gw36: exercise: 55.8 ± 20.6 control: 44.6 ± 14.0 (*p* = 0.03)	gw36: exercise: 32.4 ± 24.9 control: 21.3 ± 11.2 (*p* = 0.05)^d^	
Van Leeuwen, [41]	Examining the occurrence of fetal-maternal heart rate synchronization in which the mothers were either exercising regularly or were sedentary	maternal exercise: 57.6 ± 22.2 control: 44.9 ± 12.5 (n.s.)	maternal exercise: 35.2 ± 26.9 control: 20.8 ± 9.1 (n.s.)	---
		fetal exercise: 29.3 ± 9.6 control: 21.4 ± 6.2 (*p* = 0.01)	fetal exercise: 8.6 ± 3.8 control: 6.2 ± 2.1 (*p* = 0.02)	
May, Suminski, [37]	Correlation between continuous and non-continuous maternal LTPA and fetal HRV	fetal continuous LTPA *R* = 0.38 (*p* < 0.05)	fetal continuous LTPA *R* = 0.31 (n.s.)	---
		non-continuous LTPA *R* = 0.33 (*p* < 0.05)	non-continuous LTPA *R* = 0.04 (n.s.)	
May, Scholtz, [39]	Difference in infant HRV between exercise and control group	infant exercise: 38.8 ± 10.8 control: 34.8 ± 14.6 (n.s.)	infant^a^ exercise: 1.03 ± 0.14 control: 0.87 ± 0.21 (*p* = 0.01)	---
Gustafson, [42]	Difference in fetal HRV between exercise and control group adjusted for fetal breathing	---	fetal^a^ estimate: 0.34; SE: 0.14 (n.s.)	fetal^a^ estimate: 1.06; SE: 0.36 (n.s.)
May, [38]	Correlation between maternal LTPA (intensity, duration) and fetal HRV Regression to predict fetal heart measures	fetal intensity^b^ *R* = 0.28 (*p* < 0.05)	fetal intensity^b^ *R* = 0.27 (n.s.)	---
		duration^c^ *R* = 0.44 (*p* < 0.005)	duration^c^ *R* = 0.39 (*p* < 0.01)	
		positively predicted by intensity (*p* = 0.033)	positively predicted by duration (*p* = 0.005)	
Stutzman [43]	Within and between group changes from pre (gw20) to post (gw36) for each of the four groups; results are only presented for the HRV outcomes measured in supine position	---	---	maternal exercise – NW: gw20: 777.1 ± 1072 gw36: 787.8 ± 1042 (n.s.)
				exercise – OaO: gw20: 523.2 ± 621 gw36: 231.9 ± 203.5 (n.s.)
				control – NW: gw20: 746.3 ± 667 gw36: 461.3 ± 583 (n.s.)
				control – OaO: gw20: 652.1 ± 984 gw36: 326.1 ± 506 (sig.)^*^
May, [40]	Changes in fetal HRV between exercise and control groups depending on fetal state (active vs. passive) at gw28, gw32, and gw36	fetal^a,e^ gw28: exercise: 3.1 ± 0.3 control 2.9 ± 0.2 (n.s.)	fetal^a,e^ gw28: exercise: 1.8 ± 0.3 control 1.6 ± 0.3 (n.s.)	---
		gw32: exercise: 3.2 ± 0.3 control: 3.1 ± 0.3 (n.s.)	gw32: exercise: 1.8 ± 0.3 control: 1.6 ± 0.3 (n.s.)	
		gw36: exercise: 3.4 ± 0.3 control: 3.1 ± 0.3 (*p* = 0.03)	gw36: exercise: 2.0 ± 0.5 control: 1.7 ± 0.3 (*p* = 0.03)	
Satyapriya [36]	Changes in maternal HRV pre and during exercise session and pre and post exercise session (acute effects) at gw20 and gw36 (effects over time) Important: differences between maternal HRV measurements at gw20 and gw36 (effects over time) were not reported to be tested for significance	---	---	---

(*F* fetal, *gw* gestational week, *HRV* heart rate variability, *LTPA* leisure-time physical activity, *M* maternal, *ms* millisecond, *NW* normal weight; *n.s* not significant, *OaO* overweight and obese, *RMSSD* root mean square of successive difference, *SE* standard error, *sig* significant, *SDNN* standard deviation of normal-to-normal intervals, *TP* total power)

*p-values were not provided

^a^data were log transformed to fulfill Gaussian distribution

^b^kcal · min^−1^

^c^minutes during third trimester

^d^level of significance was defined as *p* ≤ 0.05 for all analyzes performed by Van Leeuwen et al. [41]

^e^measurements are only presented for active fetal state. Note that sample size is reduced at all time points (GA weeks 28 (*n* = 39), 32 (*n* = 37), 36 (*N* = 29)); results for quiet fetal state are not presented in this table because sample size is very small in this group

**Table 4:** Outcome measurements of each study

First author, year	Type of analyses	VLF (ms^2^)	LF (ms^2^)	HF (ms^2^)	IntF (ms^2^)
May, [47]	Difference in maternal HRV between exercise and control group at gw28, gw32, and gw36	maternal gw28: exercise: 1082.6 ± 1135.6 control: 558.9 ± 392.0 (*p* = 0.03)	maternal gw28: exercise: 436.8 ± 614.0 control: 263.7 ± 213.0 (n.s.)	maternal gw28: exercise: 1402.3 ± 2990.0 contro:l 431.1 ± 395.4 (n.s.)	---
		gw32: exercise: 1237.2 ± 942.5 control: 669.1 ± 513.1 (*p* = 0.008)	gw32: exercise: 377.2 ± 473.7 control: 186.3 ± 135.9 (*p* = 0.05)^d^	gw32: exercise: 911.2 ± 1334.8 control: 363.5 ± 365.0 (*p* = 0.048)	
		gw36: exercise: 1529.4 ± 1325.8 control: 1097.2 ± 701.9 (n.s.)	gw36: exercise: 368.6 ± 394.1 control: 201.2 ± 163.3 (n.s.)	gw36: exercise: 805.6 ± 1165.5 control: 322.4 ± 387.4 (n.s.)	
Van Leeuwen, [41]	Difference in maternal and fetal HRV between exercise and control group	---	---	---	---
May, Suminski, [37]	Correlation between continuous and non-continuous maternal LTPA and fetal HRV	fetal continuous LTPA *R* = 0.45 (*p* < 0.005)	fetal continuous LTPA *R* = 0.34 (*p* < 0.05)	fetal continuous LTPA *R* = 0.4 (*p* < 0.05)	---
		non-continuous LTPA *R* = 0.06 (n.s.)	non-continuous LTPA *R* = 0.29 (n.s.)	non-continuous LTPA *R* = 0.03 (n.s.)	
May, Scholtz, [39]	Difference in infant HRV between exercise and control group	---	infant^a^ exercise: 2.38 ± 0.2 control: 2.06 ± 0.36 (*p* = 0.002)	infant^a^ exercise: 1.72 ± 0.27 control: 1.38 ± 0.39 (*p* = 0.004)	---
Gustafson, [42]	Difference in fetal HRV between exercise and control group adjusted for fetal breathing	fetal^a^ estimate: 1.25; SE: 0.45 (n.s.)	fetal^a^ estimate: 1.01 SE: 0.46 (n.s.)	fetal^a^ estimate: 0.81; SE: 0.25 (*p* = 0.03)	fetal^a^ estimate: 1.22; SE: 0.42 (*p* = 0.03)
May, [38]	Correlation between maternal LTPA (intensity, duration) and fetal HRV Regression to predict fetal heart measures	fetal intensity^b^ *R* = 0.29 (*p* < 0.05)	fetal intensity^b^ *R* = 0.20 (n.s.)	fetal intensity^b^ *R* = 0.24 (n.s.)	fetal intensity^b^ *R* = 0.27 (n.s.)
		duration^c^ *R* = 0.34 (*p* < 0.05)	duration^c^ *R* = 0.32 (*p* < 0.05)	duration^c^ *R* = 0.38 (*p* < 0.01)	duration^c^ *R* = 0.32 (*p* < 0.05)
		positively predicted by duration (*p* = 0.007)	positively predicted by duration (*p* = 0.014)	positively predicted by duration (*p* = 0.003)	---
Stutzman [43]	Within and between group changes from pre (gw20) to post (gw36) for each of the four groups; results are only presented for the HRV outcomes measured in supine position	---	maternal exercise – NW: gw20: 194.4 ± 242 gw36: 116.2 ± 148 (n.s.)	maternal exercise – NW: gw20: 278.9 ± 504 gw36: 290.8 ± 606 (n.s.)	---
			exercise – OaO: gw20: 126.6 ± 143.7 gw36: 37.8 ± 15 (n.s.)	exercise – OaO: gw20: 101.1 ± 99.7 gw36: 25.4 ± 23.8 (n.s.)	
			control – NW: gw20: 184.2 ± 176 gw36: 83.4 ± 76 (sig.)^*^	control – NW: gw20: 361.9 ± 370 gw36: 111.8 ± 161 (n.s.)	
			control – OaO: gw20: 141.1 ± 181 gw36: 107.5 ± 202.1 (sig.)^*^	control – OaO: gw20: 376.1 ± 723 gw36: 122.2 ± 265.7 (n.s.)	
May, [40]	Changes in fetal HRV between exercise and control groups depending on fetal state (active vs. passive) at gw28, gw32, and gw36	fetal^a,e^ gw28: exercise: 4.2 ± 0.5 control 3.9 ± 0.5 (n.s.)	fetal^a,e^ gw28: exercise: 4.2 ± 0.5 control 3.9 ± 0.5 (n.s.)	fetal^a,e^ gw28: exercise: 2.2 ± 0.5 control 1.8 ± 0.5 (n.s.)	fetal^a,e^ gw28: exercise: 2.3 ± 0.6 control 1.7 ± 0.9 (n.s.)
		gw32: exercise: 4.6 ± 0.6 control: 4.1 ± 0.5 (n.s.)	gw32: exercise: 4.6 ± 0.6 control: 4.1 ± 0.5 (n.s.)	gw32: exercise: 2.2 ± 0.6 control: 1.9 ± 0.5 (n.s.)	gw32: exercise: 1.9 ± 0.8 control: 1.3 ± 0.6 (n.s.)
		gw36: exercise: 4.8 ± 0.6 control: 4.2 ± 0.6 (*p* = 0.04)	gw36: exercise: 4.8 ± 0.6 control: 4.2 ± 0.6 (*p* = 0.03)	gw36: exercise: 2.6 ± 0.8 control: 2.0 ± 0.4 (*p* = 0.01)	gw36: exercise: 2.3 ± 0.9 control: 1.4 ± 0.7 (*p* = 0.02)
Satyapriya [36]	Changes in maternal HRV pre and during exercise session and pre and post exercise session (acute effects) at gw20 and gw36 (effects over time) Important: differences between maternal HRV measurements at gw20 and gw36 (effects over time) were not reported to be tested for significance		maternal pre (gw20) and post (gw36) intervention	maternal pre (gw20) and post (gw36) intervention	---
			gw20: exercise pre session: 74.8 ± 5.9 post session: 74.1 ± 6.6 (n.s.) control pre session: 73.3 ± 8.0 post session: 70.5 ± 11.6 (n.s.)	gw20: exercise pre session: 25.2 ± 5.9 post session: 26.0 ± 6.6 (n.s.) control pre session: 26.7 ± 8.0 post session: 29.4 ± 11.6 (n.s.)	
			gw36: exercise pre session: 76.3 ± 4.9 post session: 72.5 ± 6.0 (*p* = 0.001) control pre session: 74.2 ± 9.5 post session: 70.9 ± 7.4 (n.s.)	gw36: exercise pre session: 23.2 ± 4.9 post session: 27.5 ± 6.0 (*p* = 0.001) control pre session: 25.0 ± 7.2 post session: 29.1 ± 7.4 (*p* = 0.006)	

(*F* fetal, *gw* gestational week, *HF* high frequency, *HRV* heart rate variability, *IntF* intermediate frequency, *LF* low frequency, *LTPA* leisure-time physical activity, *M* maternal, *ms* millisecond, *NW* normal weight, *n.s* not significant, *OaO* overweight and obese, *SE* standard error, *sig* significant, *VLF* very low frequency)

^*^ *p*-values were not provided

^a^data were log transformed to fulfil Gaussian distribution

^b^kcal · min^−1^

^c^minutes during third trimester

^**d**^level of significance was defined as *p* ≤ 0.05 for all analyzes performed by Van Leeuwen et al. [41]

^e^measurements are only presented for active fetal state. Note that sample size is reduced at all time points (GA weeks 28 (*n* = 39), 32 (*n* = 37), 36 (*N* = 29)); results for quiet fetal state are not presented in this table because sample size is very small in this group

**Table 5:** Outcome measurements of each study

First author, year	Type of analyses	LF/HF (ms^2^)	HF/TP (ms^2^)	VLF/LF (ms^2^)	VLF/HF (ms^2^)
May, [47]	Difference in maternal HRV between exercise and control group at gw28, gw32, and gw36	maternal gw28: exercise: 0.6 ± 0.4 control: 0.85 ± 0.6 (n.s.)	---	---	---
		gw32: exercise: 1.05 ± 0.9 control: 0.77 ± 0.5 (n.s.)			
		gw36: exercise: 1.07 ± 1.5 control: 1.2 ± 1.1 (n.s.)			
Van Leeuwen, [41]	Difference in maternal and fetal HRV between exercise and control group	---	---	---	---
May, Suminski, [37]	Correlation between continuous and non-continuous maternal LTPA and fetal HRV	---	---	---	---
May, Scholtz, [39]	Difference in infant HRV between exercise and control group	infant exercise: 5.02 ± 2.16 control: 5.23 ± 2.24 (n.s.)	---	---	---
Gustafson, [42]	Difference in fetal HRV between exercise and control group adjusted for fetal breathing	fetal^a^ estimate: 0.20; SE: 0.37 (n.s.)	---	fetal^a^ estimate: 0.24; SE: 0.41 (n.s.)	fetal^a^ estimate: 0.59; SE: 0.46 (n.s.)
May, [38]	Correlation between maternal LTPA (intensity, duration) and fetal HRV Regression to predict fetal heart measures	---	---	---	
Stutzman [43]	Within and between group changes from pre (gw20) to post (gw36) for each of the four groups; results are only presented for the HRV outcomes measured in supine position	maternal exercise – NW: gw20: 2.02 ± 1.6 gw36: 3.51 ± 3.16 (n.s.)	maternal exercise – NW: gw20: 0.217 ± 0.15 gw36: 0.187 ± 0.22 (n.s.)	---	---
		exercise – OaO: gw20: 1.66 ± 1.1 gw36: 2.08 ± 1.3 (n.s.)	exercise – OaO: gw20: 0.207 ± 0.12 gw36: 0.120 ± 0.06 (n.s.)		
		control – NW: gw20: 0.97 ± 0.92 gw36: 2.26 ± 2.1 (n.s.)	control – NW: gw20: 0.396 ± 0.16 gw36: 0.175 ± 0.09 (n.s.)		
		control – OaO: gw20: 1.3 ± 1.7 gw36: 2.18 ± 1.3 (n.s.)	control – OaO: gw20: 0.365 ± 0.19 gw36: 0.188 ± 0.19 (n.s.)		
May, [40]	Changes in fetal HRV between exercise and control groups depending on fetal state (active vs. passive) at gw28, gw32, and gw36	---	---	---	---
Satyapriya [36]	Changes in maternal HRV pre and during exercise session and pre and post exercise session (acute effects) at gw20 and gw36 (effects over time) Important: differences between maternal HRV measurements at gw20 and gw36 (effects over time) were not reported to be tested for significance	maternal pre (gw20) and post (gw36) intervention	---	---	---
		gw20: exercise pre session: 3.5 ± 0.2 post session: 3.1 ± 1.0 (n.s.) control pre session: 2.9 ± 0.8 post session: 2.8 ± 1.2 (n.s.)			
		gw36: exercise pre session: 3.5 ± 0.8 post session: 2.8 ± 0.8 (*p* = 0.001) control pre session: 3.2 ± 0.9 post session: 2.6 ± 0.9 (*p* = 0.001)			

(*F* fetal, *gw* gestational week, *HF* high frequency, *HRV* heart rate variability, *LF* low frequency, *LTPA* leisure-time physical activity, *M* maternal, *ms* millisecond, *NW* normal weight, *n.s* not significant, *OaO* overweight and obese, *sig* significant, *SE* standard error, *TP* total power, *VLF* very low frequency)

^a^data were log transformed to fulfil Gaussian distribution

^b^measurements are only presented for active fetal state. Note that sample size is reduced at all time points (GA weeks 28 (*n* = 39), 32 (*n* = 37), 36 (*N* = 29)); results for quiet fetal state are not presented in this table because sample size is very small in this group

### Study outcomes on HRV

#### Maternal outcomes

Four articles presented results concerning the influence of regular maternal PA on *maternal* HRV [36, 41, 43, 47]. May et al. [47] showed that the standard deviation of normal-to-normal intervals (SDNN), the root mean square of successive difference (RMSSD), very low frequency (VLF) HRV parameter, low frequency (LF) HRV parameter as well as high frequency (HF) HRV parameter were significantly increased in women who consistently exercised for a minimum of 30 min on three or more days (exercise group) at least at one time point of measurement in pregnancy compared to women of the control group who exercised less (detailed information see Tables 3, 4, and 5). Van Leeuwen et al. [41] showed no significant differences in maternal HRV parameters but showed a trend of increased maternal RMSSD (*p* = 0.07) in women who exercised for a minimum of 30 min on three or more days a week (exercise group) compared to women of the control group who exercised less during pregnancy.

Stutzman et al. [43] showed that total power frequency (TP) HRV parameter was significantly decreased from pre-to-post intervention only in the OaO control group. LF was also significantly decreased over time in both control groups (NW and OaO) but not in the two exercise groups (NW and OaO). HF and the ratio of LF and HF (LF/HF), as well as the ratio of HF and TP (HF/TP) showed no significant changes from pre-to-post intervention in any of the four groups.

Satyapriya et al. [36] tested maternal HRV of an exercise group which performed yoga and a control group which performed standard prenatal exercise. They assessed changes pre and post the exercise session (acute effects of PA) at gestational week 20 and gestational week 36 (intervention effect over time). At gestational week 20, no significant changes from pre-to-post exercise session were found for any HRV parameter (LF, HF, LF/HF) neither in the yoga nor in the prenatal exercise group. At gestational week 36, LF was significantly decreased from pre-to-post exercise session in the yoga group but not in the prenatal exercise group. Both groups showed a significant increase of HF from pre-to-post exercise session at gestational week 36 and a significant decrease of LF/HF from pre-to-post exercise session at gestational week 36. In summary, significant acute effects of yoga and standard prenatal exercise on HRV were only measured at gestational week 36 and not at gestational week 20 (Tables 3, 4, and 5).

#### Fetal outcomes

Five articles presented results concerning the influence of regular maternal PA on *fetal* HRV [37, 38, 40–42].

Van Leeuwen et al. [41] showed that the SDNN and the RMSSD were significantly increased in fetuses of women who exercised for a minimum of 30 min on three or more days a week (exercise group) compared fetuses of women in the control group. May, Suminski et al. [37] assessed whether fetal HRV was correlated with continuous or non-continuous maternal LTPA. The SDNN significantly correlated with both continuous and non-continuous maternal LTPA. VLF, LF, and HF were significantly correlated with continuous but not with non-continuous maternal LTPA. RMSSD did not significantly correlate with continuous nor did it correlate with non-continuous maternal LTPA. Gustafson et al. [42] estimated the difference of fetal HRV exercise in periods of fetal breathing and periods of no-fetal breathing of women who exercised for a minimum of 30 min on three or more days a week (exercise group) compared to the control group who did less exercise. RMSSD, TP, VLF, and LF were not significantly different between the exercise and control group, regardless of fetal breathing or not, but showed trends of significance (*p* = 0.08, *p* = 0.06, *p* = 0.06, and *p* = 0.07 respectively). The intermediate frequency (IntF) as well as the HF were significantly increased in fetuses of the exercise group compared to fetuses of the control group. May et al. [38] assessed whether fetal HRV is correlated with intensity (kilocalories per minute) and duration (minutes of PA during third trimester) of maternal PA performed in the third trimester of pregnancy. SDNN and VLF significantly correlated with both intensity as well as duration. The RMSSD, LF, HF, and IntF significantly correlated with duration but not with intensity. Finally, May et al. [40] assessed the difference of fetal HRV of fetuses in women who exercised for a minimum of 30 min on three or more days a week (exercise group) compared fetuses in women of the control group who did less exercise, depending on fetal state at three different time points (weeks of gestation 28, 32, and 36) in pregnancy. In active fetal state, at gestational week 36 all assessed parameters of HRV (SDNN, RMSSD, VLF, LF, IntF, HF) were significantly higher in fetuses of women in the exercise group compared to fetuses of women in the control group. At gestational week 28 and gestational week 32 differences were not significant for any of these parameters (Tables 3, 4, and 5).

#### Infant outcomes

Only one article referred to the influence of regular maternal PA on *infant* HRV [39]. The authors showed that RMSSD, LF and HF were significant higher in infants born of women who performed a minimum of 30 min of exercise three times a week (exercise group) compared to women who did less exercise (control group). SDNN and LF/HF were not significantly different between these groups (Tables 3, 4, and 5).

### Quality assessment

Using the standardized QATQS, the overall rating of the two articles referring to the intervention studies was strong [36, 43], the articles of May, Scholtz et al. [39] and May et al. [38] was moderate, and the articles of May et al. [47], Van Leeuwen et al. [41], May, Suminski et al. [37], Gustafson et al. [42], and May et al. [40] was weak (Table 6). The longitudinal study by May et al. [40] and related articles were rated weak for ‘representativeness’ since the authors provided inconsistent reporting of the sample size without providing reasons for drop-outs. In consequence, the readers of the articles are not able to retrace whether the samples of the articles are representative of the target population or not. In the intervention study performed by Satyapriya et al. [36], trainers and participants could not be blinded, but the team who did the assessments and statistics was blinded.

**Table 6:** Quality assessment of the included articles according to the EPHPP tool

First author, year	Representativeness	Design	Confounders	Blinding^b^	Methods	Drop-outs	Global rating^a^
May, [47]	Weak	Moderate	Strong	NA	Strong	Weak	Weak
Van Leeuwen, [41]	Weak	Moderate	Weak	NA	Strong	Weak	Weak
May, Suminski, [37]	Weak	Moderate	Strong	NA	Strong	Weak	Weak
May, Scholtz, [39]	Weak	Moderate	Strong	NA	Strong	Strong	Moderate
Gustafson, [42]	Weak	Moderate	Weak	NA	Strong	Weak	Weak
May, [38]	Weak	Moderate	Strong	NA	Strong	Strong	Moderate
Stutzman [43]	Moderate	Strong	Strong	NA	Strong	Strong	Strong
May, [40]	Weak	Moderate	Weak	NA	Strong	Weak	Weak
Satyapriya [36]	Moderate	Strong	Strong	NA	Moderate	Moderate	Strong

(*EPHPP* Effective Public Health Practice Project, *NA* not applicable)

^a^Strong, no weak component rating; moderate, one weak component rating; weak, two or more weak component ratings

^b^The component ‘blinding of outcome assessors and participants’ has been considered not applicable for observational and interventional studies. The reason for considering blinding not applicable for intervention studies in this case is that in studies with physical activity intervention the assessors (i.e. researchers) and the participants are very likely to know the outcome of the randomization [51]

## Discussion

This systematic review of scientific literature on the influence of maternal PA on maternal, fetal or infant HRV identified nine articles. A summary of all findings is given in Table 7. Overall, the four articles that included maternal HRV as an outcome showed inconsistent results [36, 41, 43, 47]. Of the five articles that referred to the influence of maternal PA on fetal HRV, all the articles showed increases in fetal HRV depending on maternal PA on most parameters [37, 38, 40–42].

**Table 7:** Summary of study results concerning the influence of maternal PA on HRV

Finding	First author, year	SDNN	RMSSD	TP	HF	HF/TP	IntF	LF	VLF	VLF/HF	VLF/LF	LF/HF
Maternal	May, [47]	↑	↑^a^	-	↑^a^	-	-	↑^a^	↑^a^	-	-	n.s.
	Van Leeuwen, [41]	n.s.	n.s.	-	-	-	-	-	-	-	-	-
	Stutzman [43]	-	-	(↑)	n.s.	n.s.	-	(↑)	-	-	-	n.s.
	Satyapriya [36]	-	-	-	↑	-	-	↓	-	-	-	↓
Fetal	Van Leeuwen, [41]	↑	↑	-	-	-	-	-	-	-	-	-
	May, Suminski, [37]	↑	n.s.	-	↑	-	-	↑	↑	-	-	-
	Gustafson, [42]	-	n.s.	n.s.	↑	-	↑	n.s.	n.s.	n.s.	n.s.	n.s.
	May, [38]	↑	↑	-	↑	-	↑	↑	↑	-	-	-
	May, [40]	↑^a^	↑^a^	-	↑^a^	-	↑^a^	↑^a^	↑^a^	-	-	-
Infant	May, Scholtz, [39]	n.s.	↑	-	↑	-	-	↑	-	-	-	-

(*HF* high frequency, *HRV* heart rate variability, *IntF* intermediate frequency, *LF* low frequency, *n.s. RMSSD* root mean square of successive difference, *SDNN* standard deviation of normal-to-normal intervals, *TP* total power, *VLF* very low frequency)

↑, increase; ↓, decrease; -, variable not assessed

(↑), HRV parameters were decreased from gw20 to gw36 in all 4 groups but these decreases were only significant in the control groups. In consequence, there was a relative increase of maternal HRV parameters in the exercise groups compared to the control groups namely that the decrease of HRV was smaller in the exercise groups. Therefore, we used this symbol (↑) to illustrate relative increase

^a^significant changes in HRV between exercise and control group have been found but not at all time-points of measuring (gw28, gw32, gw36)

The inconsistency of findings with regard to maternal HRV might be due to differences in PA intensity, timing in pregnancy, or due to differences in weight status of the participants. One article showed trends of increased HRV (moderate intensity exercise, small sample size) [41], one showed decreased HRV (OaO pregnant women) [43], another showed increased HRV (moderate intensity exercise) [47], and one article showed no changes on HRV (low intensity exercise group) [36]., Both, increased BMI and progressing pregnancy cause increased sympathetic control resulting in altered HRV outcomes [15–20]. It is expected that based on both of these scenarios the OaO pregnant women will have a different exercise HRV adaptation and therefore, the study performed by Stutzman et al. [43] found a decreased HRV compared to the other studies that investigated normal weight pregnant women. Additionally, moderate intensity PA was found to be associated with a significant increase [47] or a trend of increased HRV [41] whereas low intensity PA was not associated with changes in HRV [36]. This, however may lead to the conclusion that regularly performed maternal PA of moderate intensity may be more effective to improve maternal HRV than low intensity PA. To verify this hypothesis, more studies, including different intensities of maternal PA and its influence on maternal HRV, should be performed.

Results of the five articles on fetal HRV were more consistent, but the association of maternal PA with fetal HRV might change over time. The significant increases of different fetal HRV parameters presented in the article of May et al. [40] were only significant at gestational week 36 but not at weeks of gestation 28 and 32. The only article that referred to the influence of maternal PA on infant HRV reported significant increases in most of the HRV parameters [39]. Therefore, the overall consistency of the findings is high reflecting that maternal PA may increase fetal HRV, especially in late pregnancy. Nevertheless, since quality of four of five articles assessing fetal HRV was weak, and were based on only one study, level of evidence has to be rated as low.

The overall global rating for the standardized quality assessment of the articles is moderate to weak (Table 6). Only the two intervention studies were of strong quality [36, 43]. However, these articles only reported data on maternal and not on fetal or infant HRV. Furthermore, they reported inconsistent results showing decreases as well as increases of maternal HRV depending on the type of maternal exercise. In addition, Satyapriya et al. [36] compared maternal HRV between women of an exercise group that performed yoga and women of a control group that performed standard prenatal exercise. Since both groups, the exercise as well as the control group, were physically active, it is unclear whether the changes in HRV depends on PA alone or on for example pregnancy induced cardiovascular adaptations. Therefore, future intervention studies in pregnant women assessing HRV measurements depending on maternal PA should include a physically non-active control group.

Seven articles originated from the same study and sample carried out by May et al. [40]. Although the authors highlight in each article that respective data are post-hoc analyses of a subset of the original longitudinal study, it is unclear why the number of participants differs among these articles (Table 1). In the original article the authors report that 61 women were enrolled into the study and state that not all women participated in all three visits due to non-compliance or delivery prior to 36 weeks of gestation. However, the reasons for the different sample sizes reported in the studies, as well as for participant drop-out, were unclear. For studies assessing PA in participants, especially in pregnant women, it is of utter importance to clarify why participants were excluded or dropped out of a study to ensure that the reason for drop-out was not potential complications as a result of PA. Only one article [40] took fetal state (active vs. quiet) into account when assessing the influence of maternal PA on fetal HRV depending on fetal state (active vs. quiet). Since fetal heart measurements are mediated by fetal behavior [52, 53], future studies should consider this aspect. Without taking fetal state into account, interpretations of fetal HRV data are impossible. Furthermore, the authors used the MPAQ to retrospectively assess self-reported LTPA performed during the nine months of pregnancy plus three months before pregnancy. Since objective measurements of PA delivers more valid results than self-reported (subjective) measurements [54, 55], future studies assessing PA in pregnancy should use objective devices such as accelerometers, or use an exercise intervention (randomized design).

## Conclusions

Based on the current evidence available and the inconsistency of the study results, our overall conclusion is that the hypothesis that maternal PA influences maternal HRV cannot be supported. Nonetheless, some studies showed a trend that maternal HRV may be improved, especially after regularly performed maternal PA of moderate intensity.

Given that most of the articles referring to fetal HRV showed positive effects, we also conclude that there is a trend that maternal PA might increase fetal HRV. Only one study assessed infant HRV, and therefore no conclusion can be drawn as yet. As a consequence, we are not able to provide a clinical conclusion at this time but moderate intensity maternal PA might be more beneficial to improve HRV compared to low intensity maternal PA. Since HRV is a noninvasive and surrogate marker to determine fetal overall health and the development of fetal ANS, more evidence is needed to clarify whether this highly relevant marker during pregnancy can be influenced by maternal PA. Therefore, we recommend that future studies on the influence of maternal PA on HRV should consider exercise intensity, maternal overweight and obesity, progression of gestation as well as fetal state. Additionally, future studies should include well-designed intervention studies with a physically inactive control group or longitudinal studies objectively measuring maternal PA.

## Appendix 1 Complete search term for Pubmed

("Motor Activity"[Mesh:NoExp] OR "Exercise"[Mesh] OR "Sports"[Mesh] OR "Physical Exertion"[Mesh] OR "Early Ambulation"[Mesh] OR "Exercise Therapy"[Mesh] OR "Exercise Movement Techniques"[Mesh] OR “Motor Activit*”[tiab] OR “Physical Activit*”[tiab] OR “Locomotor Activit*”[tiab] OR Exercis*[tiab] OR “Physical Exercis*”[tiab] OR “Isometric Exercis*”[tiab] OR “Aerobic Exercis*”[tiab] OR Training[tiab] OR Stretching[tiab] OR “Physical Condition*”[tiab] OR “Physical Fitness”[tiab] OR “Physical Endurance”[tiab] OR “Movement Therap*”[tiab] OR “Fitness Training”[tiab] OR Plyometric[tiab] OR Stretch-Shortening[tiab] OR Weight-Lifting[tiab] OR Weight-Bearing[tiab] OR Running[tiab] OR Jogging[tiab] OR Walk*[tiab] OR Bicycle[tiab] OR Cycle[tiab] OR Bicycling[tiab] OR Cycling[tiab] OR Rowing[tiab] OR Swim*[tiab] OR Ambulation[tiab] OR Mobil*[tiab] OR Pilates[tiab] OR Yoga[tiab]) AND (Pregnancy[Mesh] OR Gravidity[Mesh] OR “Pregnant Women”[Mesh] OR Pregnan*[tiab] OR Gravid*[tiab] OR Gestation*[tiab] OR “Pregnant Wom#n”[tiab] OR Childbearing[tiab] OR Matern*[tiab] OR Fetal[tiab]) AND (“Heart Rate Variability”[Mesh] OR “Heart Rate Variability”[tw] OR “Heart Outcome”[tiab] OR “Heart Measurement”[tiab] OR “Cardiovascular Outcome”[tiab] OR “Cardiovascular Measurement”[tiab] OR “Cardiovascular Adaptation”[tiab] OR “Autonomic Nervous System”[tiab] OR “Parasympathetic Nervous System”[tiab] OR “Sympathetic Nervous System”[tiab] OR “Vagus Nerve”[tiab]).

## Abbreviations

ANS: autonomic nervous system
C: control group
E: exercise group
ECG: electrocardiograph
EPHPP: Effective Public Health Practice Project
F: fetal
gw: gestational week
HF: high frequency
HRV: heart rate variability
I: infant
IntF: intermediate frequency
LF: low frequency
LTPA: leisure-time physical activity
M: maternal
MCG: magnetocardiogram
MPAQ: Modifiable Physical Activity Questionnaire
NA: not applicable
NN: normal-to-normal
NW: normal weight
OaO: overweight and obese
RMSSD: root mean square of successive difference
SDNN: standard deviation of normal-to-normal intervals
SE: standard error
TP: total power
VLF: very low frequency
